# The Impact of Nutrition, Physical Activity, Beneficial Microbes, and Fecal Microbiota Transplant for Improving Health

**DOI:** 10.3390/life13051124

**Published:** 2023-05-02

**Authors:** Justine Keathley, Jessica White, Gregor Reid

**Affiliations:** 1 Department of Human Health and Nutritional Sciences, University of Guelph, 50 Stone Road East, Guelph, ON N1G 2W1, Canada; jkeathle@uoguelph.ca; 2 Department of Food and Nutritional Sciences, Brescia College, 1285 Western Road, London, ON N6G 1H2, Canada; 3 Departments of Microbiology & Immunology and Surgery, The University of Western Ontario, London, ON N6A 3K7, Canada; 4 Lawson Health Research Institute, 268 Grosvenor Street, London, ON N6A 4V2, Canada

**Keywords:** diet, gut microbiota, fecal microbiota transplant, probiotics, health

## Abstract

The recognition that microbes are integral to human life has led to studies on how to manipulate them in favor of health outcomes. To date, there has been no conjoint recommendation for the intake of dietary compounds that can complement the ingested organisms in terms of promoting an improved health outcome. The aim of this review is to discuss how beneficial microbes in the form of probiotics, fermented foods, and donor feces are being used to manage health. In addition, we explore the rationale for selecting beneficial microbial strains and aligning diets to accommodate their propagation in the gut. A pilot clinical trial design is presented to examine the effects of probiotics and exercise in patients with phenylketonuria (PKU); it is the most common inborn error of amino acid metabolism, and it is a complication that requires lifelong dietary intervention. The example design is provided to illustrate the importance of using omics technology to see if the intervention elevates neuroactive biogenic amines in the plasma; increases the abundance of *Eubacterium rectale, Coprococcus eutactus, Akkermansia muciniphila*, or *Butyricicoccus*; and increases *Escherichia/Shigella* in the gut, all as markers of improved health. By emphasizing the combined importance of diet, microbial supplements, and the gut microbiome, we hope that future studies will better align these components, not only to improve outcomes, but also to enhance our understanding of the mechanisms.

## 1. Introduction

The gut microbiota plays a key role in human health by degrading food, releasing important metabolites, removing or detoxifying certain compounds, and modulating host immunity. The microbiota is affected by a multitude of factors, including age, physical activity, dietary intake, and antibiotic use, among others [1,2]. In neonates, nutritional status (breastfed vs. formula fed), gestational age (term vs. preterm), and mode of delivery (vaginal vs. cesarean) have been demonstrated to have significant impacts on the gut microbiome [3]. Our understanding of the association between microbes and health is improving, but many questions still remain unanswered. The purpose of this review is to discuss how our current knowledge of beneficial microbes and health may impact future research and clinical practice in this field, and we highlight some specific studies of interest that are primarily related to nutrition and physical activity. The PubMed database was searched for relevant articles for the abovementioned purpose using keywords that are related to nutrition, physical activity, microbes, and health, and the keywords were combined with Boolean operators.

## 2. Fecal Microbiota Transplant (FMT)

FMT has been successfully employed to resolve cases of recurrent *Clostridioides difficile* (*C. difficile*) [4], including in patients with inflammatory bowel disease [5]. Donors are identified by following a rigorous investigation of their health status, with many candidates being ruled out for various reasons. The cost to identify a donor is significant; however, healthcare systems are not presently covering these expenses. Therefore, FMT is typically only offered in research settings by hospital sources and private clinics. While the absence of pathogens is a major inclusion criterion for donors, the actual composition of the microbiota and presence of certain species is currently not a factor. This is primarily because the tools have not been available or because the process has not been affordable, but it is also because there has been little evidence to suggest that this matters clinically for curing recurrent *C. difficile*.

The same FMT concept has since been applied to treat a range of conditions without altering the composition of the donor sample or by having the recipient consume a diet that is more aligned with the donor’s; this is a major concern of FMTs. For example, FMT has been used to help treat ulcerative colitis [6], non-alcoholic fatty liver disease [7], and multiple sclerosis [8], and it is even used for Sjogren Syndrome (dry mouth) and individuals with immune-mediated dry eye [9]. The etiology of each of these diseases is very different. Presumably, the hypothesis is that stool from any healthy person will contain suitable organisms that can overcome the negative impact of the recipient’s microbes. However, is this realistic?

As the composition of food intake affects microbial metabolism [10], the fecal microbiota that is present in the donor is mostly as a result of that person’s diet. However, there has been little attempt to have the recipient consume the donor’s diet post-FMT with the goal of maintaining an optimal and stable microbiota. The extent to which this failure necessitates repeat FMT treatment is not known, but a recent study has demonstrated that this may be less of a concern than originally anticipated. Specifically, in a small cohort of 13 individuals who had received FMT to treat recurrent *C. difficile,* 80% of the pre-FMT strains in the recipient were eliminated 5 years post-FMT despite no attempt to align the diet with that of the donor [11]. This demonstrates promise for the development and use of defined live biotherapeutic products for the treatment of recurrent *C. difficile* infection and suggests that future research in this area would be beneficial.

Other approaches to modulate the gut microbiome have included probiotic organisms, which are defined as “live microorganisms that, when administered in adequate amounts, confer a health benefit on the host” [12]. However, too rarely have the strains been selected with characteristics that are suitable for the issues that the target host faces, such as the ability to modulate transit time, decrease inflammation, produce certain neurotransmitters, or enhance anti-oxidant activity [13,14,15,16,17]. Furthermore, probiotic organisms with specific traits have so far not been added to FMTs for the purposes of improving health outcomes; this is an area that warrants future investigation.

## 3. Aging and the Gut Microbiome

The more research that is reported on the gut microbiota, the better appreciation we have for factors that influence its composition and function. These studies have implications for how donors are selected for FMTs. For example, a large study of over 1000 Chinese people aged from youths to centenarians [18] showed that the microbiota of the centenarian cohort was remarkably similar to people over the age of 30 years, suggesting that the maintenance of a health-promoting gut microbiota through life is feasible. Therefore, age per se may not be as big a factor as diet and living in the same location throughout life. This being the case, there may be organisms within the gut of centenarians that are important for longevity; if these were identified, FMT donors could include a cohort having these strains or include people over 30 years whose gut microbiota contains these strains.

If organisms are to be selected for administration to the gut, can microbiome studies of healthy elderly persons be insightful? The increased abundance of *Akkermansia* reduces the presence of *Faecalibacterium*, *Bacteroidaceae*, and *Lachnospiraceae* with aging [19], which may help to identify FMT criterion or strains that could be transplanted [20]. The findings of one study showed how a Mediterranean diet can alter the gut microbiome in the elderly, resulting in more short/branch-chained fatty acids and lower toxic compounds, thereby decreasing markers of frailty and inflammation and increasing cognitive function [21]; the findings indicate that diet can result in the identification and propagation of beneficial strains. These and other studies [22] make it potentially feasible to direct the gut microbiome in favor of health as well as to increase the pool of people that are able to make donations for FMTs.

## 4. The Microbiota Gut-Brain Connection and Physical Activity

Strenuous and intense physical activity, especially in the heat, can decrease gut permeability and increase inflammatory responses [23]. This depends on the type of activity, as demonstrated in an interventional study where insulin-resistant participants were randomized to perform sprint intervals or moderate-intensity, continuous training; these training sessions resulted in beneficial gut microbial changes. Specifically, a decreased ratio of Firmicutes/Bacteroidetes, *Clostridium*, and *Blautia and an* increased Bacteroidetes were observed two weeks after the training [1]. In a related study of the gut microbiota of Finnish cross-country skiers at the end of an exhausting training and competitive season, there was a reduction in the abundance of several mucin-degrading organisms, including *Akkermansia muciniphila*; however, a healthier serum lipid profile was observed in these participants when compared with physically active controls [24]. Specifically, *Butyricicoccus* was positively associated with high-density lipoprotein (HDL) cholesterol, HDL2 cholesterol, and HDL particle size; this association with an altered gut microbial profile in that study [24] illustrates that while the athletes were fit and would theoretically have their feces be suitable for transplant, this would not be appropriate for a recipient with cardiovascular issues. However, the standard criteria for selecting a fecal donor would not take into consideration this correlation. Regardless of whether or not the athletes altered their diets compared with the controls, the study also illustrates that exercise can alter the microbiota.

Although the results from rodent studies are not directly applicable to human health, they may be used to generate hypotheses for future human clinical trials. One rodent study showed that the production of endocannabinoid metabolites from the gut microbiota increased dopamine in the ventral striatum and further resulted in improved running performance [25]. The paper, like many that equate the gut with brain effects [26], assumes that bacteria in the gut, not the oral cavity, urogenital tract, or other areas of the mouse, caused the effect. The paper identified *Eubacterium rectale* and *Coprococcus eutactus* as being important for the improved performance. The authors inoculated single species into germ-free mice to prove that the animals could run faster. While this experimental approach is not directly applicable to humans, we might hypothesize that we would observe similar findings in human studies; however, the authors failed to prove that these species are prevalent in Olympic or other elite athletes, as would be expected [25]. The findings also contradict those of Motiani et al. [1], which showed that physical activity reduced the abundance of Firmicutes, of which *E. rectale* and *C. eutactus* are phylum members.

Endurance athletes are known to take a range of ergogenic aids, some of which include minerals and chemical compounds that are known as zeolites, with the aim of immune stimulation. However, a study of 52 endurance athletes showed that 12 weeks of supplementation lowered amounts of the gut barrier protein zonulin [27]. In a smaller study, male athletes receiving a six-strain probiotic for 14 weeks showed an improvement in gut barrier function as well as a decrease in the pro-inflammatory marker tumor necrosis factor alpha (TNF-α) when compared with the placebo [28]. Another study using competitive cyclists and triathletes and a single probiotic strain, *Lactobacillus fermentum* PCC^®^, showed a lower severity of gastrointestinal symptoms [29]. However, can prebiotic and probiotic intake influence athletic performance? Research in this area is promising [30], but more clinical trials are needed in order to inform clinical practice guidelines for athletes. Adherence to the definition of prebiotics (“a substrate that is selectively utilized by host microorganisms conferring a health benefit” [31]) and using appropriate prebiotic amounts are important.

The Dohnalova study [25] also proposed that motivation is important for exercise and that microbes somehow affect the striatum and dopamine receptors. Notably, the authors showed a rapid and sustained upregulation of dopamine in the ventral and dorsal striatum after exercise. To further probe the mechanisms, the authors exposed isolated dorsal root ganglia neurons in vitro to mouse stool extracts and found that N-oleoylethanolamide stimulated activity. The authors suggested the new term “interoceptomimetics”, which they define as “molecules that stimulate afferent sensory pathways and thereby influence brain activity by peripheral intervention”. The idea is that these molecules could motivate people to exercise. Experimentally, the approach has not only reductionist problems because of its testing of neurons in vitro, but it fails to prove that *E. rectale* and *C. eutactus* within a fecal biofilm can cause increased vascular dissemination of specific molecules that would then affect the striatum. Not only that, but others have suggested that in order to address obesity, rather than focusing on expending more calories, we should focus on attempting to make the brain crave less food. An example of this comes from a food-craving study in pregnancy that found an association with key components of the dopaminergic mesolimbic circuit, namely with the upregulation of nucleus accumbens (NAc), dopamine receptor 2 (Drd2) expression, and activity of D2R neurons [32]. As food craving has long-lasting effects on offspring, such as glucose intolerance, obesity, and anxiety disorders even into adulthood, the study suggests that rather than having *E. rectale* and *C. eutactus* be present in the gut to promote exercise, it would be better if they were absent, given that they might increase food-craving. Such conflicting data makes it difficult to know which microbial intervention is worth pursuing. Further human clinical trials are needed in this area.

## 5. Nutrition and the Gut Microbiome

As gut microbes rely on the food we ingest for replication and retention, it is no surprise that diet has a significant effect on microbiota composition, structure, and function [33,34]. In a 2014 study [35], an animal-based diet decreased the levels of Firmicutes, which metabolize dietary plant polysaccharides (*Roseburia, E. rectale*, and *Ruminococcus bromii*). However, following on from the previous discussion, these findings beg the question: what should someone eat prior to an exercise regimen: food that increases or decreases the abundance of organisms such as *E. rectale*?

The Mediterranean diet has been mentioned above. Another option was explored in an effort to manipulate the gut microbiota using fermented foods. It should be noted that fermented foods (defined as “foods made through desired microbial growth and enzymatic conversions of food components” [36]) are not probiotic and do not have probiotics in them unless specifically added and documented. The 17-week, randomized, prospective study was performed using plant-based fiber and fermented foods [37]. The protocol for the fermented foods included six servings per day of kombucha, yogurt, kefir, buttermilk, kvass (6 oz), kimchi, sauerkraut, other fermented veggies (1/4 cup), and/or a vegetable brine drink (2 oz). This shows that the authors were not basing the desired outcome on specific microbes, but instead hoping that beneficial microbes in general would meet the primary outcome of changing the cytokine response score within each arm from baseline (−2 weeks prior) to the end of the maintenance phase (week 10). The authors note limitations in the causality and mechanisms, but nevertheless found that inflammatory markers were decreased, and that microbial diversity increased in people consuming the fermented food diet. Given the large variability of food types and organisms within them, some might argue that it is difficult to draw conclusions that would lead to dietary guidelines or an explanation for any recommendation, except that a variety of fermented foods have apparent immunological benefits. Others, however, might argue that the approach used by Wastyk et al. [37] was highly pragmatic given that in a real-world setting, individuals are free to choose from a variety of food products and typically do not follow a strict intervention protocol, which is limited in variety.

An issue of potential importance, not only for the gut but also for the brain, is how sensory elements play a role. The texture, taste, appearance, and smell of food as well as its shelf-life are influential in what people consume and how their body responds to it. Oftentimes, people “eat with their eyes”, as foods will be judged first before tasting it [38]. The food industry has cleverly crafted foods using salt, sugar, fats, additives, and the removal of water, which are often inexpensive and appealing to the senses but are nutritionally imbalanced [39]. Additives have long been used to influence these sensory elements. One recent review of carrageenan additives (sulfated polysaccharides from seaweed and red algae) that are used as thickening and gelling agents as well as in cosmetics and hygiene products explored the potential negative effects on the gut microbiota [40]. Among the mechanisms, carrageenans can attenuate digestive proteases; disturb intestinal barrier proteins such as zonulin-1 (Zo1); may reduce the thickness of the gut mucus layer; increase interleukin-8 (IL-8) expression, nuclear factor kappa light chain enhancer of active B cells (NF-kB) activation, and reactive oxygen species in colonic epithelial cells; and decrease bacterial richness. The latter coincides with the reduction of metabolites such as butyrate. The net effect of inadequately digested proteins is for them to be fermented by the colonic microbiota, leading to the production of toxic metabolites, such as hydrogen sulfide, indole, and ammonia [41]. Notably, the rate of the global use of carrageenans is rapidly increasing [42].

On the other hand, the intake of 250 mg of carrageenans for twenty days has also been shown to reduce total cholesterol and low-density lipoprotein cholesterol levels in patients with hypercholesterolemia (*p* < 0.05). Does this mean that carageenans could be added to a cholesterol-lowering probiotic strain [43], and if so, how would its effects compare to a statin? The latter drugs are the mainstay of the cardiovascular management of cholesterol, but they have significant side effects and act through cytochrome P450, meaning that they can interfere with other pharmaceutical agents [44]. There is no evidence to date of probiotic strains or carrageenans having such drug interactions. This would undoubtedly be an interesting future research endeavor.

Interestingly, the claim from rodent studies that non-nutritive sweeteners negatively impact the gut microbiota [45] has not been verified in clinical trials. A recent 14-day intervention study showed no changes in gut bacteria when human participants consumed a dose that was equivalent to three 355 mL cans of diet beverage each day [46]. In a small human study, an emulsifier, carboxymethylcellulose, which is added to foods to improve texture and increase shelf life, was shown to reduce gut microbiota diversity and be associated with lower levels of short-chain fatty acids and free amino acids [47]. More research is warranted to determine if specific food additives could benefit or harm the gut microbiome.

Beyond food and nutrient consumption, the smell of foods should also be considered when exploring the gut microbiome. The smell of foods influences taste, desire, and craving [48], but how does this relate to the microbiome? A study of the nasal microbiota showed differences that were associated with three olfactory functions (odor threshold, discrimination, and identification) [49]. Interestingly, butyric acid producers were associated with impaired olfactory function. As such, it is reasonable to suspect that this may influence a person’s diet and their gut microbiome, as smell shapes perception and eating behavior, as well as mood, memories, and social interactions.

Overall, it is clear that diet alters the gut microbiome, and studies have been uncovering dietary interventions to retain gut functionality. One review concluded that a balanced diet containing saturated and monosaturated fatty acids, microbiota-accessible carbohydrates, protein, phytochemicals, vitamins, and minerals along with limited n-6 polyunsaturated fats, simple carbohydrates, food-derived bioactive peptides, and iron could contribute to the restoration of intestinal homeostasis [50]. The very nature of this exhaustive and somewhat non-specific list of recommended nutrients makes it difficult to apply to general populations. However, some studies are now evaluating the impact of single foods on the gut microbiota. For example, a recent human interventional study of mango pulp consumption demonstrated an association for both cardiovascular outcomes and enhanced gut microbial diversity with the abundance of some bacterial species [51]. Additionally, the overall health benefits associated with consuming a Mediterranean diet have been well established. A pilot study investigated the consumption of a Westernized fast-food diet versus a Mediterranean diet for four days. After consuming the fast-food, the composition of the gut closely resembled what has been associated with chronic disease versus the Mediterranean diet, which showed the opposite [52].

In terms of high protein intake, the toxic metabolites of branch-chained fatty acids, ammonia, indoles, phenols, amines, and hydrogen sulfide increase the risk of cardiovascular and intestinal diseases as well as obesity, type 2 diabetes mellitus (T2DM), and central nervous system (CNS) diseases [53]. Unless countered by probiotics and prebiotics that increase short-chain fatty acids, skeletal muscle loss can occur from protein anabolism in the elderly [53,54]. Several studies have demonstrated that plant-based diets can optimize health via the promotion of gut microbial diversity and stable microbial systems. This is likely a result of higher fiber intake leading to increased lactic acid bacteria and enhanced presence of short-chain fatty acids [55]. Perhaps a plant-based diet coupled with probiotics and prebiotics is optimal for aging?

Further research is needed to confirm or refute these associations and attempt to personalize which substances an individual should consume (and in what amounts) for good health.

As research continues to progress, it is possible that dietitians and other nutrition care specialists will be able to provide more specific nutrition recommendations for optimizing the gut microbiota in the future.

## 6. FMT and Probiotic Strain Alignment with Host Health Status

Following on from the pioneering work of Allen-Vercoe in trying to select strains from the feces of a healthy donor and propagating them to replace FMTs [56], a number of artificial FMTs have been developed [57]. This work has been driven by a desire for reproducibility and to avoid the issues of identifying and retaining access to donor stool, both of which are time-consuming and expensive [58]; furthermore, this work seeks to sift out strains that may have pathogenic potential or have no role to play in colonizing the recipient and out-competing *C. difficile*.

One such product, SER-109, still requires donor stool, which is then processed through a proprietary system to remove vegetative bacteria, fungi, parasites, and viruses. The end result is a range of organisms that includes *Bacillus, Clostridium, Eubacterium, Blautia*, and *Roseburia* along with many others [59,60]. The therapy has been demonstrated to reduce the recurrence of infection, which could have potentially occurred through bile acid production that inhibited the pathogen’s spores. Interestingly, the product is not referred to as a probiotic, which was perhaps done to avoid comparison to existing probiotics and make an easier path through the Food and Drug Administration (FDA) for approval; alternatively, it is because the strains are not documented and differ between each batch, thus not meeting the probiotic definition [12].

A company that was developing another neuroprotective compound, CP101, cancelled the product trials, while Ferring Pharma and NuBiyota have continued their development; the latter company has managed to grow strict anaerobes and encapsulate them into high quality drugs with optimal stability, with the intent of first treating recurrent *C. difficile* [61]. The details of these company products are not yet divulged due to intellectual property issues, but presumably, the strains have been selected because they can co-exist, be safely applied, and interfere with infection. To the best of our knowledge, their administration does not come with any dietary recommendations.

As with many probiotic strains and products, the success of preventing or treating one condition often leads to the strains and products being tested for other diseases. For example, *Lactcaseibacillus* (formerly *Lactobacillus*) *rhamnosus* GG was initially shown in a yogurt formulation to have an effect at preventing antibiotic-associated diarrhea [62]; then, the strain was used in a dried form to prevent atopic dermatitis in infants [63]. The same approach has been taken with FMTs as noted by the broadening range of conditions that it is being used for. What has yet to be explored, however, are the health impacts of adding probiotic strains to fecal matter or selecting FMTs based on their metabolites.

If better disease management outcomes occur from the consumption of dietary factors that improve the function of FMTs or probiotics in the recipient, how would this be measured? One way would be to examine different fecal samples using multiple labeling for different genera to identify associations, co-dependencies, and biofilm structures [64]. For example, if *Akkermansia* is present in low abundance it may propagates when acetate is consumed [65]; or when folate precursors are ingested, folate-producing bifidobacteria could proliferate [66]. Culturing could be used to show whether an added *Lactobacillus* probiotic multiplied, and gas chromatography/mass spectroscopy (GC/MS) could measure altered metabolic readouts [67,68].

## 7. Future Human Clinical Intervention Trials: The Example of Pku

**Figure 1:**
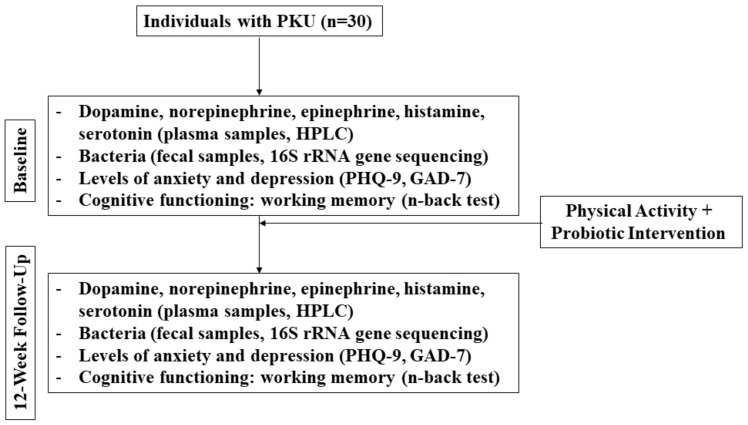
Flow diagram of mock clinical trial in PKU patients.

It is apparent that more research, specifically more human interventional studies, is urgently needed in this field in an effort to inform clinical applications. While clinical resources have been developed (based on human interventional studies) to guide probiotic use to improve the outcomes that are related to irritable bowel syndrome, constipation, and *C. difficile,* among others (see for example: probioticchart.ca), it is biologically plausible that probiotics could improve outcomes for a variety of conditions that may not have as obvious of a connection as gastrointestinal-related conditions; thus, they have yet to be investigated. For example, studies are demonstrating that the gut microbiome may have a significant effect on the development and pathogenesis of neurological disorders [69]. The following is a brief example of a novel human pilot study that could be envisaged to assess some of the points raised above (Figure 1).

Phenylketonuria (PKU) is the most common inborn error of amino acid metabolism. This condition is characterized by reduced enzymatic functioning, primarily of phenylalanine hydroxylase (PAH); this results in a lack of conversion of L-phenylalanine (Phe) to L-tyrosine (Tyr) and thus leads to a build-up of Phe in the blood and, subsequently, the brain [70]. The pathophysiology underlying cognitive impairment in PKU is attributed to the accumulation of neurotoxic Phe metabolites and a deficiency in the uptake of large neutral amino acids (LNNAs) leading to an impairment of protein and neurotransmitter biosynthesis [71]. Without following a strict diet that is limited in Phe, permanent intellectual disability, seizures, and behavioral problems occur. Nerve cells in the brain are especially sensitive to Phe concentrations, thus the permanent brain damage when levels are too high [72]. While gene editing may eventually repair or delete defective genes in such diseases [73], other approaches warrant investigation.

There are five biogenic amine neurotransmitters: serotonin, dopamine, norepinephrine (noradrenaline), histamine, and epinephrine (adrenaline). They can be impaired due to inhibition of Tyr and tryptophan hydroxylases and competition with amino acids at the blood–brain barrier. Adults with PKU have decreased levels of 5-hydroxyindoleacetic acid (5-HIAA); 5-hydroxytryptophan (5-HTP), which correlates with precuneus and frontal atrophy, respectively; and reduced availability of serotonin and dopamine in the brain [74].

Exercise can help activate the cerebellum, occipital lobe, parietal lobe, and frontal lobe of Parkinson’s Disease (PD) patients [75]. While exercise and ingestion of beneficial microbes are not able to prevent or treat PKU, there may be a rationale for them contributing to its management by improving motor and non-motor symptoms and by reducing oxidative stress and inflammation [76].

In the following mock study, the hypothesis is that an intervention of daily exercise and ingestion of certain beneficial microbes for 12 weeks will result in increased neuroactive biogenic amines in the plasma; increased abundance of *E. rectale, C. eutactus, A. muciniphila*, or *Butyricicoccus*; and increased abundance of *Escherichia/Shigella* in individuals with PKU.

### Selection of Intervention

The choice of the microbial intervention herein is based upon two considerations. Firstly, because fermented foods have variable concentrations of organisms and because some can have undesirable biogenic amines (putrescine, tyramine, cadaverine, and histamine) [77] as well as gamma-aminobutyric acid (GABA), serotonin, dopamine and other neuroactive compounds [78], we decided there were too many confounders to choose these foods. Instead, we selected two probiotic products to be taken together.

The first is *Escherichia coli* Nissle 1917, which is a probiotic with anti-inflammatory properties. As there is a reported negative association with PD and abundance of *Escherichia/Shigella* [79], the study will determine if there is an increase in *Escherichia/Shigella* abundance and, secondarily, a decrease in any of the genera (*Clostridium IV, Aquabacterium, Holdemania, Sphingomonas, Clostridium XVIII, Butyricicoccus*, and *Anaerotruncus*) associated with PD [80].

The second is *Lactocaseibacillus rhamnosus* GG, which is a strain that is able to produce GABA [81,82], reduce inflammation [83], and modulate the gut microbiota [84]. It is also commercially available, as is *E. coli* Nissle 1917.

Because this is an open-label, single arm, pilot study, 20 individuals with PKU will be recruited and baseline measurements will be compared with week 12.

Participants will be asked to exercise each day in whatever manner meets their lifestyle, but they must reach at least 10,000 daily steps. No probiotic beyond the intervention or fermented food products that are prescribed will be permitted during this study. Dietary recalls will be collected to assess the possible intake of Phe and foods that may confer prebiotic effects and contain carrageenans and other additives. Participants will self-collect stool samples each day and track the number of bowel movements as an indicator of transit time. This will help to laterally evaluate if certain foods have an effect on the fecal microbiota composition. Validated questionnaires will be used to assess mental health and cognitive function as secondary outcomes (e.g., Patient Health Questionnaire (PHQ-9), Generalized Anxiety Disorder Questionnaire (GAD-7), n-back test).

To determine the primary outcome of the changes in dopamine, norepinephrine, epinephrine, histamine, and serotonin, a high-performance liquid chromatography (HPLC) analysis will be used on the plasma samples [85], while 16S rRNA gene sequencing of fecal samples will identify bacteria to the species level [68,86].

If the hypothesis is true, the results of this study would demonstrate positive changes in the neuroactive amines, gut microbiota composition, and cognitive function when comparing the baseline observations with those from week 12. The study is a pilot from which a sample size for a larger clinical trial can be determined.

## 8. Conclusions

**Figure 2:**
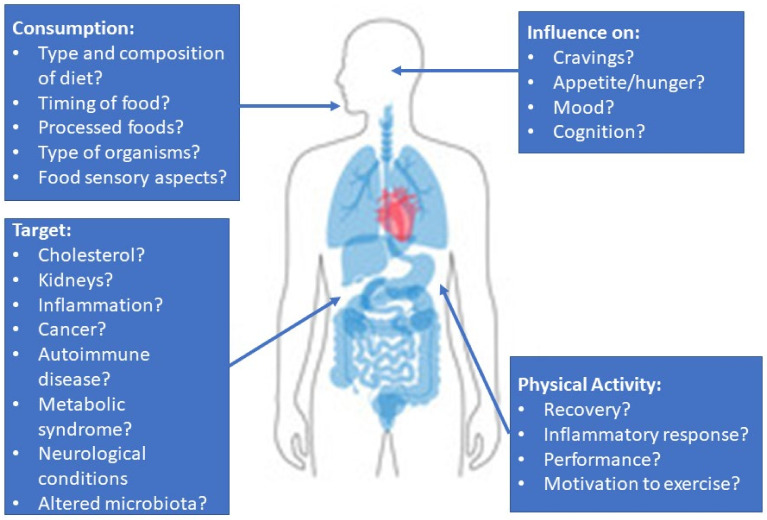
A summary of the issues relevant to the study of microbes on human health and topics of interest for clinical studies.

The link between the nutrients consumed and how the microbiota react to them is slowly being appreciated. However, human interventional studies are needed to test specific microbes (probiotic or FMT compositions) with a dietary intake that encourages the growth of desired organisms and metabolites for each individual’s primary health issue. These could be markers that are associated with the cardiovascular system, brain, liver, or pancreas (Figure 2). In potentially fatal diseases such as PKU and *C. difficile*, aligning microbes with diet could further improve disease management and patient prognosis. With greater research in this field, the hope is that clinical practice guidelines will eventually be able to recommend specific microbes to improve a variety of diseases/conditions.

## Data Availability

Not available.

## Abbreviations

**Abbreviation**	**Definition**
5-HIAA	5-hydroxyindoleacetic acid
5-HTP	5-hydroxytryptophan
*A. muciniphila*	*Akkermansia muciniphila*
*C. difficile*	*Clostridioides difficile*
*C. eutactus*	*Coprococcus eutactus*
CNS	Central nervous system
Drd2	Dopamine receptor 2 gene
*E. coli*	*Escherichia coli*
*E. rectale*	*Eubacterium rectale*
FDA	Food and Drug Administration
FMT	Fecal microbiota transplant
GABA	Gamma-aminobutyric acid
GAD-7	Generalized Anxiety Disorder Questionnaire
HDL	High-density lipoprotein
HPLC	High-performance liquid chromatography
IL-8	Interleukin-8
LNNA	Large neutral amino acids
Nac	Nucleus accumbens
NF-kB	Nuclear factor kappa-light-chain enhancer of activated B cells
PAH	Phenylalanine hydroxylase
PD	Parkinson’s disease
Phe	L-phenylalanine
PHQ-9	Patient Health Questionnaire
PKU	Phenylketonuria
T2DM	Type 2 diabetes mellitus
TNF-α	Tumor necrosis factor alpha
Tyr	L-tyrosine
Zol-1	Zonulin-1
